# Impact of Maternal Daily Oral Low-Dose Vitamin A Supplementation on the Mother-Infant Pair: A Randomised Placebo-Controlled Trial in China

**DOI:** 10.3390/nu13072370

**Published:** 2021-07-10

**Authors:** Ye Ding, Ping Hu, Yue Yang, Fangping Xu, Fang Li, Xiaolong Lu, Zhencheng Xie, Zhixu Wang

**Affiliations:** Department of Maternal, Child and Adolescent Health, School of Public Health, Nanjing Medical University, Nanjing 211166, China; dingye@njmu.edu.cn (Y.D.); huping950208@163.com (P.H.); byyangyue@163.com (Y.Y.); m18356581510_1@163.com (F.X.); LiFang7208@163.com (F.L.); lu123xiaolong@163.com (X.L.); zhenchengxie@njmu.edu.cn (Z.X.)

**Keywords:** Vitamin A supplement, lactating mothers, serum, breast milk, infant

## Abstract

Background: The nutritional status of vitamin A in lactating mothers and infants is still not optimistic. Due to the dietary habits and dietary restrictions of postpartum customs in China, vitamin A supplementation has been advocated as a potential strategy to improve vitamin A status of lactating mothers with inadequate dietary vitamin A intake. Existing clinical trials are limited to single or double high-dose maternal administrations. However, in China, vitamin A supplements are readily available in the form of daily oral low-dose supplements, and the effect of these is unknown. This study aimed to evaluate the effects of daily oral low-dose vitamin A supplementation on the retinol levels in the serum and breast milk of lactating mothers and the health status of infants in China. Methods: Lactating mothers who met the inclusion criteria and planned to continue exclusive breastfeeding were randomly assigned to receive either daily oral vitamin A and D drops (one soft capsule of 1800 IU vitamin A and 600 IU vitamin D_2_), or a matching placebo for 2 months. Before and after the intervention, dietary intake was investigated by instant photography, and the retinol concentration in maternal serum and breast milk was determined by ultra-high performance liquid chromatography-tandem mass spectrometry. During the trial, the health status of infants was diagnosed by a paediatrician or reported by lactating mothers. A total of 245 participants completed the study, with 117 in the supplementation group and 128 in the control group. Results: After the 2-month intervention, maternal serum retinol concentrations increased in the supplementation group with no change in the control group. Although breast milk retinol concentrations decreased significantly in both groups, the decrease in the supplementation group was significantly lower than that in the control group. However, maternal vitamin A supplementation was not associated with a lower risk of infant febrile illness, respiratory tract infection, diarrhoea, and eczema. Conclusions: Daily oral low-dose vitamin A supplementation is helpful in improving maternal vitamin A status, despite having no effect on infant health status through breast milk.

## 1. Introduction

Vitamin A is an essential nutrient that is important for maintaining normal vision, gene expression, reproduction, embryonic development, growth, and immune function [1]. The human body cannot synthesise this nutrient, which is obtained through the diet [1]. When dietary intake is low for a long time, vitamin A deficiency (VAD), defined as a serum retinol concentration of less than 0.70 μmol/L [2], will occur. This deficiency remains a global public health problem. Compared with adults in non-special physiological period, lactating mothers and infants need more vitamin A and could face a greater risk of VAD. In China, the serum retinol concentration of about 2.96 million (17.90%) woman of childbearing age [3] and 9.08 million (59.26%) infants aged 0.5–2 years are in the state of VAD or marginal VAD (MVAD meaning a low vitamin A concentration status), defined as a serum retinol concentration of between 0.70 μmol/L and 1.05 μmol/L, which was also estimated to signal people who might have inadequate vitamin A intake [2]. Although the data on vitamin A levels in infants within 6 months of age are relatively limited, this is enough to show that the vitamin A nutritional status of lactating mothers and infants in China is not ideal.

Although the Chinese Nutrition Society established new dietary guidelines in 2016, which emphasise increasing the intake of vitamin A-rich foods to improve the nutritional status of lactating mothers [4], a dietary survey of Chinese lactating mothers completed by our research group in 2018 showed that the dietary vitamin A intake of this population is still lower than the recommended value, especially in rural areas [5]. There are two dietary forms of vitamin A. Preformed vitamin A (retinol and retinyl ester) is derived from animal sources such as animal liver, meat, dairy products, and fish. Provitamin A (beta-carotenoid) is derived from colourful fruits and vegetables [1]. Therefore, it is very important to encourage lactating mothers to eat foods rich in vitamin A. However, if a lactating mother does not eat these foods due to their dietary habits and confinement customs after birth, vitamin A status may only be available through other forms of supplementation. In China, it is relatively easy to obtain nutrient supplements. Vitamin A supplements are viewed as an efficient and convenient way to achieve the greatest reductions in the prevalence and consequences of VAD [6]. However, it was reported that only a small number of lactating mothers used nutrient supplements, including low doses of vitamin A (563, 1350, 1500, or 2400 IU) per day compared to pregnant women [7,8]. Even in urban areas, the proportion of lactating mothers using multivitamin supplements is only 3.1%, and in rural areas, the proportion of this population is even lower [9]. To date, many studies have evaluated the effect of vitamin A supplementation in lactating mothers, but they all involve single or double high-dose maternal administrations (single doses of 200,000 IU, 300,000 IU, or 400,000 IU) [10,11]. It has not been reported whether daily low-dose vitamin A supplementation can improve the nutritional status of vitamin A in lactating mothers in China, but this is very important, which is an urgent problem to be solved.

In the general population, serum retinol levels are an indicator of vitamin A nutritional status. However, serum concentration of retinol is homeostatically regulated and remains constant under a wide range of vitamin A intake or liver stores. Thus, a substantial decrease in serum retinol concentration may not be apparent unless liver reserves of vitamin A are dangerously low [12]. Pregnancy and lactation can also affect circulating retinol concentrations. For mothers in the exclusive breastfeeding stage, in addition to serum retinol levels, breast milk retinol levels can also be used as an indicator of vitamin A status in both lactating mothers and infants [13]. Even if the mother is well nourished during pregnancy, in order to avoid the adverse reactions caused by high concentrations of retinol, only half of vitamin A in maternal serum is transferred to the foetus through the placenta [14]. The level of vitamin A in neonatal cord blood is less than 1/2 of that in maternal blood, and the infant has very little retinol reserves at birth (approximately 5 μmol) [15]. Breast milk is the only source of vitamin A in exclusively breastfed infants until complementary foods are added in the first six months of life [16]. Therefore, vitamin A in breast milk is essential to meet the needs of the baby and liver accumulation before weaning. Previous studies have shown that breastfeeding infants fed with vitamin A-deficient lactating mothers are susceptible to VAD, resulting in xerophthalmia, anaemia, and weakened iron metabolism, growth retardation, increased infectious morbidity, suppressed immune response, increased risk of respiratory tract infections, and diarrhoea-related mortality [17]. Therefore, it is necessary to study whether daily low-dose vitamin A supplementation can affect the retinol levels in the serum and breast milk of lactating mothers and the health status of infants.

## 2. Materials and Methods

### 2.1. Participants

From November 2018 to August 2019, lactating mothers at 30–45 days postpartum underwent their first postpartum examination at the Maternal and Child Health and Family Planning Service Centre of Jiangning District in Nanjing city. Among them, mothers aged 20–40 years were invited for screening if they had delivered full-term (gestational age ≥37 weeks) singleton healthy infants, had been continuously successfully lactating, and planned to continue exclusive breastfeeding for at least 2 months. Mothers with metabolic diseases such as hypertension and diabetes (including gestational diabetes), clinically diagnosed nutritional disorders (anaemia, osteoporosis, iodine deficiency goitre, et al.), mastitis and other breast diseases, disability, mental disorders, infectious diseases, any weakening or debilitating condition, or history of alcohol consumption, drug abuse, and smoking were not recruited. Infants with febrile illness, respiratory tract infection, intestinal infection, milk malabsorption, allergy, and other diseases or those receiving drug treatments were also excluded. In addition, we excluded mothers who were involved in other studies on food restriction, nutrition intervention, drug intervention, and those who were currently taking dietary supplements. All lactating mothers provided written informed consent. The trial was approved by the Ethics Committee of Nanjing Medical University and registered at http://www.chictr.org.cn/index.aspx (ChiCTR 1800020179) (accessed on 19 December 2018).

### 2.2. Study Design

This study was a parallel-group, single-blind, randomised, placebo-controlled trial. Participants who met the inclusion criteria were randomly assigned to either the supplementation group, in which they received daily oral vitamin A and D drops (one soft capsule of 1800 IU vitamin A and 600 IU vitamin D_2_, Zhejiang Hailisheng Pharmaceutical Co., Ltd., China), or a control group, in which they received placebo capsules of similar appearance that were continued daily for a period of 2 months. Participants were blinded to group assignment during the trial, while the trial personnel were aware of it.

Baseline characteristics of both groups were obtained through questionnaire interviews and confirmed by maternal and infant physical examination manual and clinical records, including maternal age, maternal height and weight at enrolment and at the end of the trial, maternal education level, gestational age, parity, delivery way, maternal medical history, maternal and infant clinical medication, and infant month age, body length, weight, and head circumference at enrolment and at the end of the trial.

All participants were required to provide a three-day dietary record, 2 mL blood samples, and 10–15 mL breast milk samples at the time of enrolment and at the end of the trial, respectively. The dietary record of lactating mothers was completed by instant photography, and the retinol concentration in maternal serum and breast milk was determined by ultra-high-performance liquid chromatography-tandem mass spectrometry (UPLC). During the trial, the health status of infants was diagnosed by a paediatrician and/or reported by lactating mothers, including the incidence of febrile illness, respiratory tract infections, diarrhoea, and eczema.

### 2.3. Dietary Data Collection, Calculation, and Evaluation

All participants were asked to complete two 3-day dietary records by instant photography within one week after enrolment and one week before the end of the trial. As previously reported, instant photography, developed by our team, is more convenient and accurate than the 24-h dietary recalls [18]. Specifically, before each meal, the food (rice, vegetables, soup, etc.) of lactating mothers should be put into flat tableware separately to ensure that they are not mixed with the food of other family members. The food was then placed in the red area of a two-dimensional background (scale with 1 cm × 1 cm). A note was suggested to put on the background next to the food, indicating the name of the food and the ingredients of the food mix. After that, the food should be photographed from directly above, from the front at 45 degrees, and from the back at 45 degrees, and the whole red area of background should appear in photographs. After the meal, the same method was used to photograph the unfinished food. Finally, food photos of lactating mothers were sent to the assessors. Food photos were compared with the food atlas of instant photography to estimate the intake of each food [19]. Primary data obtained from the food photos were entered into the EpiData software by two trained assessors to verify the accuracy. Daily food consumption, energy, and nutrient intake were calculated according to the China Food Composition Tables (6th edition) [20]. Data on energy and nutrient intake were imported into Microsoft Excel for the statistical analysis. Based on the 2013 Chinese Dietary Reference Intakes [21], the measurements of dietary vitamin A were based on the recommended nutrient intake (RNI).

### 2.4. Sample Collection and Retinol Concentration Detection

Maternal blood and milk samples were collected at the Maternal and Child Health and Family Planning Service Centre at enrolment and at the end of the trial. For the evaluation of serum retinol, 2 mL of venous blood from lactating mothers was collected into vacutainer tubes and transported to the laboratory under protection from light, where the serum obtained was separated by centrifugation and frozen at −70 °C until analysis. For the evaluation of breast milk retinol, 10–15 mL of breast milk from lactating mothers was collected between 07:30 and 09:00 a.m. with no preference for one breast, into polypropylene tubes protected from light, carried to the laboratory, and frozen at −70 °C until analysis. All participants had an automatic breast pump available.

Serum samples were treated with ethanol to precipitate proteins and then extracted twice with hexane. Retinyl acetate (Sigma Chemical Corp., St. Louis, MO, USA) dissolved in ethanol was used as an internal standard to determine the extraction efficiencies. The hexane layers were pooled, evaporated to dryness with nitrogen, and then redissolved in 200 μL of methanol: methylene dichloride (3:1, *v*/*v*). After homogenization, breast milk samples were saponified with alcohol and potassium hydroxide, washed with 100 g NaCl/L, and extracted in hexane. Retinyl acetate was then added, and the extract was washed with water and dissolved in methanol. Retinol concentrations were then determined by UPLC (Agilent 1290) by a third-party testing agency (Suzhou Harmony Health Medical Diagnostics Co., Ltd., Suzhou, China), as described previously [2,22]. This method has an intra-assay coefficient of variation (CV) of 5%. Retinol concentrations were expressed in μmol/L.

The Chinese Nutrition Society recommended that the adequate intake of vitamin A for infants aged 0–6 months should be 300 retinol activity equivalents (RAE)/d [21]. Since the breast milk intake of infants at this age is about 750 mL, we calculated that the concentration of retinol in breast milk should be at least 1.39 μmol/L. If the concentration is lower than that standard, it is defined as a low retinol concentration in breast milk. The standard of serum retinol was the concentration of MVAD (≤1.05 μmol/L). We then used these two standards to judge whether vitamin A in maternal serum and breast milk at different stages met the needs of lactating mothers and their infants.

### 2.5. Quality Control

The questionnaire used to obtain the baseline characteristics was reviewed and revised by experts to ensure its accurate relevance. All researchers were well trained on the technical aspects of the trial before the participants were recruited. Researchers explained to the participants how to record the diet and provided the participants with the introduction of instant photography, including a description of the method and the video of the specific operation. The responses to baseline characteristics and dietary information were retrieved and reviewed over time. The errors were corrected to ensure the integrity and validity of the results. For example, if there was no cereal or fruit in one day’s diet, we would ask and verify. If a lactating woman really forgot to record, we would ask her to complete another day’s dietary record again. Blood and breast milk samples were collected in strict accordance with the standard sampling methods and were transported from the hospital to a −70 °C refrigerator in the laboratory using a low-temperature storage box. The same instrument and the same batch of reagents were used to detect the retinol concentrations in serum and breast milk. The primary data were double entered into the Epidata software by two trained researchers to verify the accuracy. In the statistical analysis stage, the appropriate statistical methods were selected strictly according to the statistical requirements and data types, and the influence of potential confounding factors was controlled by multivariable analysis.

### 2.6. Sample Size and Statistical Analysis

At present, the literature on the effect of low-dose vitamin A intervention on breast milk composition in China is relatively limited. Therefore, we mainly made sample estimations based on whether the dietary vitamin A intake of Chinese lactating mothers reached the RNI level of vitamin A. As the intake of dietary vitamin A fluctuates greatly in the population, it is not suitable to calculate the sample size by means and standard deviations, but rather by rate. Therefore, the sample size was calculated using the following formula:

$$n_{1}=n_{2}=\frac{{\left[u_{\alpha }\sqrt{2\pi_{c}\left(1-\pi_{c}\right)}+u_{\beta }\sqrt{\pi_{1}\left(1-\pi_{1}\right)+\pi_{2}\left(1-\pi_{2}\right)}\right]}^{2}}{{\left(\pi_{1}-\pi_{2}\right)}^{2}}$$

According to the survey data of dietary vitamin A intake of lactating mothers 2–4 months after delivery in three cities, which were similar to Nanjing in terms of geographical location and economic level [7], the effective rate in the control group was 0.136. We assumed that after intervention with vitamin A supplements, the effective rate in the supplementation group was three times higher than that in the control group (0.408). By substituting *α* = 0.05 and *β* = 0.1 into the formula, the minimum sample size per group contained 54 participants. Since lactating mothers need to recover their body and take care of their babies, the rate of loss of follow-up is high. Assuming that 20% of the subjects might lose to follow-up during the follow-up period, the sample size of each group was adjusted to *n*_1_ = *n*_2_ = 65.

Statistical analysis of all data was performed using the Statistical Package for the Social Sciences version 22. There was a statistically significant difference at level of 0.05 (*p* < 0.05). Normally distributed continuous variables were expressed as the mean and standard deviation (SD); non-normally distributed continuous variable data were expressed as median and interquartile range (IQR). Categorical variables were expressed as frequencies (*n*) and percentages (%). An independent samples *t*-test or the chi-square (*χ*^2^) test were used to compare the normally distributed continuous variables and categorical variables related to baseline characteristics in the two groups, respectively. The Mann–Whitney U test was used to compare the differences in energy and nutrient intake between the two groups. At the time of enrolment and at the end of the trial, an independent samples *t*-test was used to analyse the differences in retinol concentrations in maternal serum and breast milk between the two groups, while the paired *t*-test was used to analyse the intragroup differences before and after the intervention. The associations between vitamin A intervention and retinol concentrations in maternal serum and breast milk were examined using multiple linear regressions, while the associations between vitamin A intervention and health status of infants were examined using logistic regression. The covariates considered mainly included maternal age, BMI at the end of trial, education, gestational weeks, parity, delivery way, dietary vitamin A intake, diet composition (fat: carbohydrate ratio), dietary energy, and protein and fibre intake, as well as infant age, weight, length, and head circumference. 

## 3. Results

### 3.1. Participants and Baseline Characteristics

**Figure 1:**
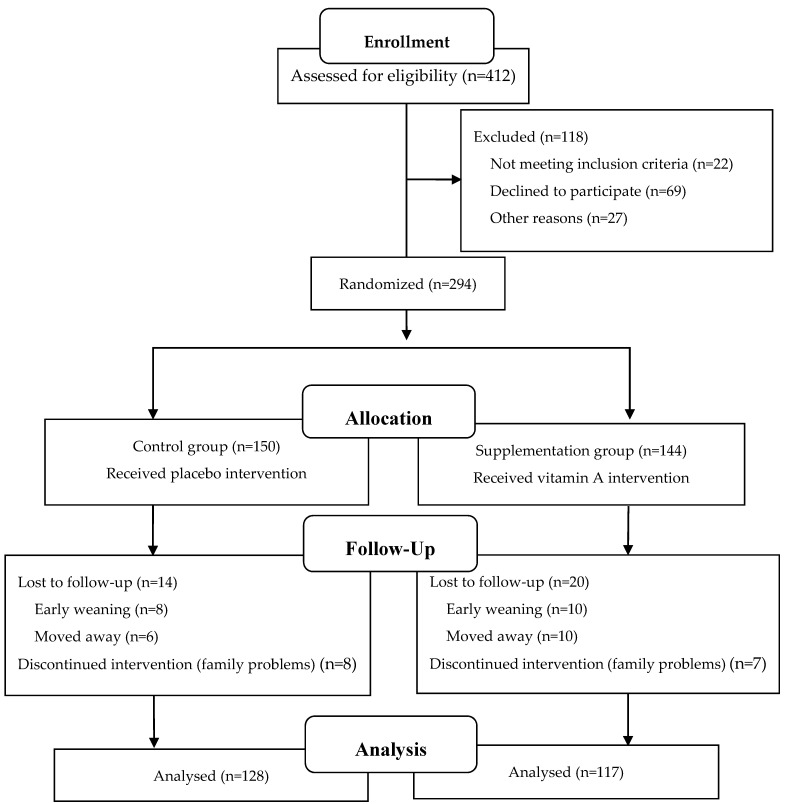
Flow diagram of the final sample for the analysis.

The participant flowchart is shown in Figure 1. A total of 412 participants were screened for eligibility, and 294 participants met the inclusion criteria and were randomly assigned to the supplementation (144 participants) and control (150 participants) groups. By the end of the trial, 245 participants completed the study (117 in the supplementation group and 128 in the control group). During the two-month intervention period, 15 participants stopped intervention due to family problems and 34 participants were lost to follow-up, of which 18 were due to early weaning and 16 were due to moving away. After statistical analysis, there was no significant difference in baseline characteristics between all subjects who completed the study and those who lost the follow-up. The differences in baseline characteristics of subjects who were lost the follow-up between the two groups were further compared, the results also showed that there was no significant difference.

The baseline characteristics of both groups completing the trial are presented in Table 1. The mean maternal age was 29.8 years old, and the mean maternal BMIs at enrolment and the end of the trial were 23.5 and 23.6 kg/m^2^, respectively. Most of them had good education, with an average of 15.66 years. The mean gestational age of these participants was 39.2 weeks, 65.7% were primiparas, and 59.6% had spontaneous labour. The characteristics of the participants were similar between the two groups, except for the maternal BMI at the end of the trial.

### 3.2. Maternal Daily Dietary Energy and Nutrient Intakes between Supplementation and Control Groups at Enrolment and the End of the Trial

In general, Table 2 shows that no significant variation was observed in dietary energy and nutrient intake between the supplementation and control groups at enrolment and at the end of the trial. Specifically, at the time of enrolment, the median dietary energy intake of supplementation and control groups were 2189.9 kcal/d and 2210.2 kcal/d, respectively. The dietary intake of energy-producing nutrients (carbohydrate, fat, and protein) was also similar between the two groups. After calculation, the fat to carbohydrate ratio was 0.30 in the supplementation and 0.34 in control groups, and the difference was not statistically significant. Moreover, in supplementation and control groups, the intake of dietary vitamin A was 711.5 µg RAE/d and 732 µg RAE/d, respectively. Compared with the RNI value of vitamin A, the number of participants whose dietary vitamin A intake was lower than the RNI value was 77.8% and 82.0%, respectively, and the difference was not statistically significant. At the end of the trial, the median dietary energy intakes were 2019.5 kcal/d and 1978.8 kcal/d in the supplementation and control groups, respectively. After the two-month period, the dietary intake of energy-producing nutrients (carbohydrate, fat, and protein) and the fat to carbohydrate ratio remained similar between the two groups. The median dietary intake of vitamin A was still comparable between the supplementation and control groups (706.6 μg RAE/d vs. 783.9 μg RAE/d), and the dietary vitamin A intake of 82.1% and 85.9% participants was below the RNI value, respectively.

### 3.3. Effect of Vitamin A Supplementation on Maternal Serum Retinol Concentration at the End of the Trial

**Figure 2:**
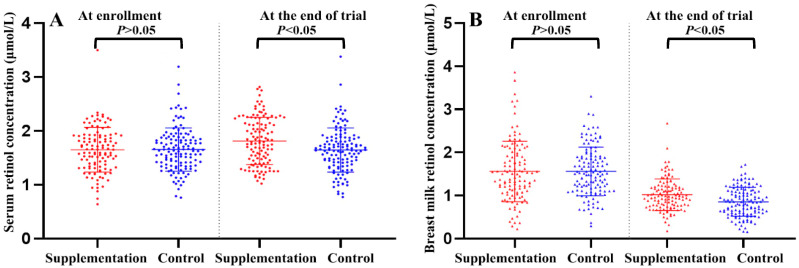
Comparison of retinol concentration in maternal serum (**A**) and breast milk (**B**) between supplementation and control groups at enrolment and the end of the trial. Values are Means ± SDs. *p*-values were assessed using Student’s *t* test.

As shown in Figure 2A, at the time of enrolment, the baseline maternal serum retinol concentrations in the supplementation and control groups were comparable with 5.3% and 4.7% of the participants having a serum retinol concentration ≤1.05 μmol/L (defined as MVAD), respectively. After the 2-month intervention, the maternal serum retinol concentration increased significantly in supplementation group (from 1.65 ± 0.41 μmol/L to 1.81 ± 0.44 μmol/L; *p* *< 0*.01) with no change in control group (from 1.66 ± 0.40 μmol/L to 1.66 ± 0.41 μmol/L; *p* = 0.98). Meanwhile, the MVAD rate decreased significantly with vitamin A supplementation (from 6.0% to 0.9%; *p* = 0.03), with no change in the placebo (from 4.7% to 8.6%; *p* = 0.21). The difference in serum retinol concentration before and after the intervention was then calculated, and the results showed that the difference between the two groups was statistically significant, accompanied by a significant difference in MVAD between the two groups.

As shown in Table 3, the maternal serum retinol concentration increased significantly in the supplementation group compared to that in the control group (*β* = 0.170, 95% CI = 0.060–0.274, *p <* 0.01). In the multivariable analysis that was adjusted for baseline characteristics (maternal age, BMI at the end of trial, education, gestational weeks, parity, and delivery way), and factors that may have affected vitamin A status, including dietary vitamin A intake and diet composition (fat: carbohydrate ratio), differences between the two groups in changes in the maternal serum retinol concentration remained significant. In addition, the intakes of dietary energy, protein, and fibre were also considered on the basis of the previous model, and they did not alter the results (data not shown).

### 3.4. Effect of Vitamin A Supplementation on Maternal Breast Milk Retinol Concentration at the End of the Trial

As shown in Figure 2B, at the time of enrolment, the baseline maternal breast milk retinol concentrations in the supplementation and control groups were comparable with 47.9% and 42.2% of participants having a breast milk retinol concentration < 1.39 μmol/L (defined as low retinol concentration in breast milk), respectively. After the 2-month intervention, breast milk retinol concentrations decreased significantly in both the supplementation (from 1.56 ± 0.70 μmol/L to 1.02 ± 0.37 μmol/L; *p* < 0.01) and control (from 1.56 ± 0.56 μmol/L to 0.85 ± 0.34 μmol/L; *p* < 0.01) groups. Meanwhile, the proportion of mothers with low retinol breast milk also increased significantly in both supplementation (from 47.9% to 85.5%; *p* < 0.01) and control (from 42.2% to 93.8%; *p* < 0.01) groups. The difference in breast milk retinol concentration before and after the intervention was then calculated, and the results showed that the decrease in the supplementation group was significantly less than that in the control group (−0.53 ± 0.69 μmol/L, vs. −0.71 ± 0.63 μmol/L; *p* < 0.01), accompanied by the significant difference of low retinol breast milk between the two groups.

As shown in Table 3, the maternal breast milk retinol concentration increased significantly in the supplementation group compared to that in the control group (*β* = 0.144, 95% CI = 0.064–0.223, *p* < 0.01). In the multivariable analysis that was adjusted for baseline characteristics (maternal age, BMI at the end of trial, education, gestational weeks, parity, and delivery method), and factors that may have affected vitamin A status, including dietary vitamin A intake and diet composition (fat: carbohydrate ratio), differences between the two groups in changes in the maternal breast milk retinol concentration remained significant. In addition, the intakes of dietary energy, protein, and fibre were also considered on the basis of the previous model, and they did not alter the results (data not shown).

### 3.5. Effect of Maternal Vitamin A Supplementation on the Health Status of Infants during the Trial

During the 2-month follow-up period, the incidence of febrile illness, respiratory tract infection, diarrhoea, and eczema in infants in both groups were analysed. The incidence rates of these diseases in the supplementation group were 8.5%, 7.7%, 6.8%, and 12%, respectively, and those in the control group were 5.5%, 14.1%, 10.9%, and 9.3%, respectively. Statistical analysis of the data showed that maternal vitamin A supplementation was not associated with lower risks of infant febrile illness (RR = 1.62, 95% CI = 0.59–4.39, *p* = 0.35), respiratory tract infection (RR = 0.61, 95% CI = 0.31–1.23, *p* = 0.16), diarrhoea (RR = 0.60, 95% CI = 0.24–1.48, *p* = 0.27), and eczema (RR = 0.90, 95% CI = 0.41–1.99, *p* = 0.80), respectively. As shown in Table 4, in the multivariable analysis that was adjusted for infant age, weight, length, and head circumference, and differences between groups in changes in risks of infant diseases (febrile illness, respiratory tract infection, diarrhoea, and eczema) remained non-significant.

## 4. Discussion

This randomised placebo-controlled trial is the beginning of a trial conducted in China in which low-dose daily vitamin A intervenes lactating mothers and explores its impact on maternal vitamin A status and the health status of infants. The results showed that low-dose vitamin A intervention significantly increased the serum retinol concentration of lactating mothers and alleviated the decrease in breast milk retinol concentration. However, maternal low-dose vitamin A supplementation was not associated with a lower risk of infant diseases (febrile illness, respiratory tract infection, diarrhoea, and eczema).

Before evaluating the effectiveness of vitamin A supplementation, it was necessary to compare dietary vitamin A levels. Previous studies mostly used 24 h dietary recalls to obtain dietary vitamin A intake information [23,24], while our study used instant photography to record the 3-day dietary intake of lactating mothers. Our previous study found that, compared with the conventional 24-hour dietary recalls, instant photography can obtain food consumption and nutrient intake data closer to the data obtained by the weighing method [18]. Therefore, the results for dietary vitamin A are more accurate. Our study showed that more than 80% of the 245 lactating mothers in Jiangning District of Nanjing city did not reach the Chinese RNI value at 30–50 days and 90–110 days after delivery. This result was similar to that of a previous survey of 537 lactating mothers in Beijing, Suzhou, and Guangzhou. Among them, 86.4% had a lower vitamin A intake than the RNI value [7]. However, our previous studies on urban and rural areas in 13 provinces and municipalities showed that more than 90% of Chinese lactating mothers had lower dietary vitamin A intake than the RNI value in 2018 [5]. The reasons for this difference may be the inconsistency of dietary survey methods, the limited dietary sources of vitamin A, and the influence of geography, customs, and economic conditions.

Previous studies have shown that different forms of vitamin A intervention can improve maternal serum retinol concentrations in lactating mothers. For example, a high-dose vitamin A intervention trial in Brazil showed that compared with the control group, the intervention of 200,000 IU of vitamin A on the 20th and 30th day after delivery significantly increased the serum retinol concentration (1.17 ± 0.34 μmol/L vs. 1.02 ± 0.28 μmol/L) and reduced the VAD prevalence (3.2% vs. 16.7%) in lactating mothers at 3 months postpartum [25]. The daily intervention trials so far were about provitamin A, mainly β-carotene. β-carotene can be converted into retinol in vivo, but its absorption and conversion rates are low. According to a previous study, the equivalent of 1 mg retinol would be 12 mg β-carotene for fruit and 28 mg β-carotene for green leafy vegetables [26]. Studies in Bangladesh and Vietnam found a significant increase in serum retinol concentrations of lactating mothers who received daily β-carotene-rich foods (42 mg or 12 mg, 5.6 mg or 4.8 mg) compared to controls [10,26,27]. However, if a lactating mother does not eat fruit and vegetables due to their dietary habits and confinement customs after birth, β-carotene status may only be available through the supplementation. A previous study found that daily supplementation with β-carotene (7.8 mg) did not improve the serum retinol concentration in lactating mothers [28]. Therefore, we used low-dose vitamin A supplementation in the form of retinol, which has a high absorption rate and can effectively reduce the safety risk of high-dose vitamin A supplementation. The common form of vitamin A supplementation in China is combined with vitamin D, and the dose of vitamin A for adults is 1800 IU. We found that the daily intervention with this dose of vitamin A for 2 months significantly increased the serum retinol concentration of lactating mothers and decreased the MVAD rate, which was similar to the results of previous studies mentioned above.

During lactation, vitamin A from dietary sources can be actively transported to the mammary gland through chylomicrons and secreted into breast milk. Vitamin A in breast milk should be maintained at an appropriate level to ensure adequate vitamin A supply in the first six months of life for infants who are exclusively breastfed and to prevent possible clinical problems. Vitamin A status in breast milk of participants involved in our study was not ideal, especially at the end of the trial, mainly because dietary vitamin A intake was low and the concentration of vitamin A in the breast milk decreased with the progress of lactation, which is similar to the results of other studies [29]. At present, the effects of different forms of vitamin A intervention on maternal breast milk retinol concentrations are inconsistent. A number of studies in Bangladesh, Indonesia, and Vietnam have shown that a single high-dose vitamin A supplementation (200,000 IU), weekly vitamin A (7000 μg or 4800 μg) supplementation, and daily or weekly supplementation of β-carotene supplementations, β-carotene, or β-cryptoxanthin-fortified foods, as well as animal and plant foods rich in vitamin A, have increased the concentration of retinol in breast milk [10,26,27,28,30]. Consistent with these findings, our study found that 1800 IU of vitamin A intervention per day for 2 months could reduce the decrease in retinol concentration in the breast milk of lactating mothers. At the end of the trial, the retinol concentration in the breast milk of the supplement group was significantly higher than that of the control group. However, some studies in Ghanaian [31] and Brazil [32] have found that vitamin A intervention has no effect on retinol concentration in breast milk. This may be due to the different forms and doses of vitamin A supplements. Both studies were conducted in the form of multivitamins, with a low dose of 800 μg retinol equivalent. The supplement time was also different; one of the trials was from the beginning of pregnancy to the end of labour. Moreover, the baseline maternal serum retinol concentration was high before the start of supplementation, which may also lead to ineffective results.

Surprisingly, we did not find an association between maternal low-dose vitamin A supplementation and lower risks of infant diseases (febrile illness, respiratory tract infection, diarrhoea, and eczema). At present, there are few studies on the effect of maternal vitamin A supplementation on the health status of infants. One study assessed the effect of two doses of 200,000 IU vitamin A intervention in lactating mothers on the incidence rate of infant diseases, including febrile illness, respiratory tract infection, and diarrhoea, but not eczema, and the results were consistent with our findings [33]. Although vitamin A enhances immunity, the immunity of infants within six months after birth is mainly guaranteed by the mother through breast milk. Colostrum is rich in immune factors, and the prebiotics in breast milk are also conducive to the growth of intestinal probiotics [34,35,36]. This may be one of the reasons why vitamin A intervention does not affect the health of infants. In addition, the coexistence of multiple micronutrient deficiencies in breast milk may be another reason. During the 2-month follow-up period, a small number of infants in both groups developed febrile illness, respiratory tract infection, diarrhoea, and eczema. Our study found that the dietary intake of water-soluble vitamins (vitamin B_1_, vitamin B_2_, vitamin B_9_, and vitamin C) and minerals (calcium, magnesium, and iodine) in lactating mothers was low (data not shown). The content of water-soluble vitamins, iodine, and other nutrients in breast milk is closely related to the diet of lactating mothers. Almost all the water-soluble vitamins needed by infants come from breast milk [37,38]. If mothers lack these nutrients, the related deficiency symptoms quickly appear in infants. Further testing of micronutrients in breast milk is needed to confirm our hypothesis.

However, our study had several limitations. First, since the common form of vitamin A supplementation in China is combined with vitamin D, the intervention group not only took vitamin A, but also took vitamin D, which is a regulatory factor for immunity and skin barrier functions. A previous case-control study in children reported that vitamin D might have a positive effect on eczema in infants [39]. Therefore, we have to admit that this may be a confounder. Second, although most of the breast milk we collected was foremilk, there were some exceptions; that is, when milk from one breast was not enough, milk from other breast was collected. As the concentration of vitamin A in hindmilk had been reported to be significantly higher than that in foremilk [40]; thus, the variability of vitamin A content in collected milk may be another confounder. Third, there was no significant difference in maternal BMI between the two groups at enrolment, but at the end of trial, that difference was statistically significant, which was beyond our control. We took this factor into account when we analysed the results and made adjustments.

## 5. Conclusions

In conclusion, supplementation of lactating mothers with daily oral low-dose vitamin A (1800 IU) for 2 months was found to have a positive impact on maternal serum and breast milk vitamin A status, but no effect on infant health status was detectable 2 months after maternal supplementation. Due to ethical constraints, we did not obtain blood from infants to determine their serum retinol concentrations at baseline and 2 months after the intervention. However, the results of this study are sufficient to show that daily supplementation of regular doses of vitamin A to lactating mothers is helpful to improve the maternal vitamin A status, despite having no effect on infant health status through breast milk. This result also provides a theoretical basis for dietary guidance for lactating mothers in China; that is, in addition to adding vitamin A-rich foods, regular doses of vitamin A supplements are also an option for lactating mothers with inadequate vitamin A intake.

## Figures and Tables

**Table 1:** Comparison of baseline maternal characteristics between supplementation and control groups.

Characteristics	Supplementation (*n* = 117)	Control (*n* = 128)	*p*-Value
Age, years	30.1 ± 4.1	29.5 ± 3.4	0.16
BMI at enrolment, kg/m^2^	23.48 ± 2.90	23.28 ± 2.49	0.34
BMI at the end of trial, kg/m^2^	24.03 ± 2.7	23.04 ± 2.4	0.03
Education, years	15.44 ± 2.99	15.22 ± 3.63	0.58
Gestational age, weeks	39.30 ± 1.27	39.49 ± 1.25	0.24
Primiparous, n (%)	73 (62.4)	88 (68.8)	0.30
Spontaneous labor, n (%)	62 (53.0)	84 (65.6)	0.05

Values are Means ± SDs for continuous variables or number and percentage (n (%)) for categorical variables. *p*-values were assessed using Student’s *t*-test or *χ*^2^ test. Supplementation group: in the form of 1800 IU retinol; control group: in the form of placebo. BMI: body mass index.

**Table 2:** Comparison of maternal daily dietary energy and nutrient intakes between supplementation and control groups at enrolment and the end of trial.

Energy and Nutrients	Supplementation (*n* = 117)	Control (*n* = 128)	*p*-Value
At Enrolment			
Energy (kcal/day)	2189.9 (1878.1, 2620.8)	2210.2 (1943.9, 2586.8)	0.74
Carbohydrate (g/day)	264.8 (224.8, 325.9)	258.8 (224.8, 314.5)	0.94
Fat (g/day)	85.0 (64.5, 97.4)	83.0 (67.2, 101.4)	0.27
Fat to carbohydrate ratio	0.30 (0.24, 0.39)	0.34 (0.23, 0.40)	0.34
Protein (g/day)	102.8 (87.4, 128.9)	102.3 (81.3, 114.6)	0.94
Vitamin A (µg RAE/day)	711.5 (521.6, 1143.3)	732 (502.5, 1078.7)	0.56
At the end of trial			
Energy (kcal/day)	2019.5 (1754.3, 2312.4)	1978.8 (1701.6, 2229.7)	0.50
Carbohydrate (g/day)	245.9 (191.1, 295.9)	248.1 (200.0, 285.3)	0.65
Fat (g/day)	69.8 (53.4, 88.1)	73.4 (54.2, 83.4)	0.27
Fat to carbohydrate ratio	0.29 (0.24, 0.39)	0.28 (0.22, 0.35)	0.95
Protein (g/day)	83.67 (73.96, 108.2)	81.94 (68.1, 101.5)	0.94
Vitamin A (µg RAE/day)	706.6 (567.4, 1097.6)	783.9 (477.6, 1066.9)	0.77

Values are median (P25, P75). *p*-values were assessed using Mann–Whitney U test. Supplementation group: in the form of 1800 IU vitamin A; control group: in the form of placebo. RAE: retinol activity equivalent.

**Table 3:** Association between maternal vitamin A supplementation and retinol concentration in maternal serum and breast milk at the end of the trial.

Dependent Variables and Models	R^2^	*β*	*β* (95% CI)	*p*-Value
Serum retinol concentration, µmol/L				
Model 1	0.038	0.170	0.060–0.274	<0.01
Model 2	0.507	0.168	0.087–0.249	<0.01
Model 3	0.508	0.171	0.089–0.252	<0.01
Breast milk retinol concentration, µmol/L				
Model 1	0.053	0.146	0.077–0.254	<0.01
Model 2	0.319	0.144	0.064–0.223	<0.01
Model 3	0.323	0.147	0.068–0.277	<0.01

Model 1: maternal vitamin A supplementation (yes/no) was the independent variable, and the other variables were not adjusted. Model 2 was adjusted for maternal age, BMI at the end of the trial, education, gestational weeks, parity, delivery way, dietary vitamin A intake, and diet composition (fat: carbohydrate ratio). Model 3 was adjusted for maternal age, BMI at the end of the trial, education, gestational weeks, parity, delivery way, dietary vitamin A intake, diet composition (fat: carbohydrate ratio), and dietary energy intake. *p*-values were assessed using a multivariable linear regression model for differences between groups with adjustment for covariates. CI: confidence interval.

**Table 4:** Association between maternal vitamin A supplementation and the health status of infants during the trial.

Disease Patterns	Supplementation (*n* = 117)	Control (*n* = 128)	RR (95% CI)	*p*-Value ^1^	Adjusted RR (95%CI)	*p*-Value ^2^
Febrile illness	10 (8.5)	7 (5.5)	1.62 (0.59–4.39)	0.35	1.63 (0.59–4.52)	0.35
Respiratory tract infection	9 (7.7)	18 (14.1)	0.61 (0.31–1.23)	0.16	0.60 (0.29–1.22)	0.16
Diarrhea	8 (6.8)	14 (10.9)	0.60 (0.24–1.48)	0.27	0.59 (0.29–1.22)	0.26
Eczema	14 (12.0)	12 (9.3)	0.90 (0.41–1.99)	0.80	0.87 (0.39–1.95)	0.73

Values are number and percentage (n (%)) unless otherwise specified. *p*-values were assessed using a multivariable logistic regression model for differences between groups, with adjustment for covariates.^1^ *p*-values were for RR; ^2^ *p*-values were for adjusted RR. Adjusted RR was adjusted for infant age, weight, length, and head circumference. RR: relative risk, CI: confidence interval.

## Data Availability

The data presented in this study are available on request from the corresponding author.
